# Role of STAT3 in the pathogenesis of nasopharyngeal carcinoma and its significance in anticancer therapy

**DOI:** 10.3389/fonc.2022.1021179

**Published:** 2022-10-13

**Authors:** Yishimei Si, Jinjing Xu, Linghan Meng, Yuanqing Wu, Jianwei Qi

**Affiliations:** Department of Otolaryngology, Nanjing Medical University Affiliated Nanjing Hospital, Nanjing, China

**Keywords:** stat3, NPC, IL-6/JAK1/STAT3, phytochemicals, Radiotherapy

## Abstract

Nasopharyngeal carcinoma (NPC) is a type of head and neck tumor with noticeable regional and ethnic differences. It is associated with Epstein-Barr virus infection and has a tendency for local and distant metastasis. NPC is also highly sensitive to radiotherapy and chemotherapy. Over 70% of patients present with locoregionally advanced disease, and distant metastasis is the primary reason for treatment failure. A signal transducer and activator of transcription 3 (STAT3) promotes NPC oncogenesis through mechanisms within cancerous cells and their interactions with the tumor microenvironment, which is critical in the initiation, progression, and metastasis of NPC. Further, p-STAT3 is strongly associated with advanced NPC. Recent research on STAT3 has focused on its expression at the center of various oncogenic pathways. Here, we discuss the role of STAT3 in NPC and its potential therapeutic inhibitors and analogs for the treatment and control of NPC.

## 1 Introduction

### 1.1 Nasopharyngeal carcinoma

Nasopharyngeal carcinoma (NPC) is a disease with regional and ethnic differences, and Southeast Asia and Mongolia currently have the highest incidence rates. According to the International Agency for Research on Cancer, approximately 129,000 new cases of NPC were recorded in 2018, with over 70% occurring in southern China and Southeast Asia (1). Epstein-Barr virus infection, environmental variables, and genetic factors are vital in the development of NPC (2). NPC cells are highly prone to local invasion of lymph nodes and distant metastases (3). Among the 87,000 new cases diagnosed annually in southern China, over 70% are locally advanced NPC (4). Moreover, 10–20% of patients with NPC develop local and/or nodular recurrence after initial treatment, leading to poor prognoses. Therefore, exploring novel therapeutic targets for NPC is critically important (5).

According to the World Health Organization, NPC is divided into keratinizing squamous, non-keratinizing squamous, and basaloid squamous subtypes. Differentiated and undifferentiated tumors are classified as non-keratinizing NPC, accounting for over 97% of NPC cases in southern China and Southeast Asia. Keratinizing NPC is more prevalent in western nations (approximately 75%) (6–8). The Epstein-Barr virus is associated with 95% of non-keratinized NPC (9, 10). These unique races, regions, pathological features, viruses, and other factors indicate the complexity of the pathogenesis of NPC.

### 1.2 STAT3

**Figure 1:**
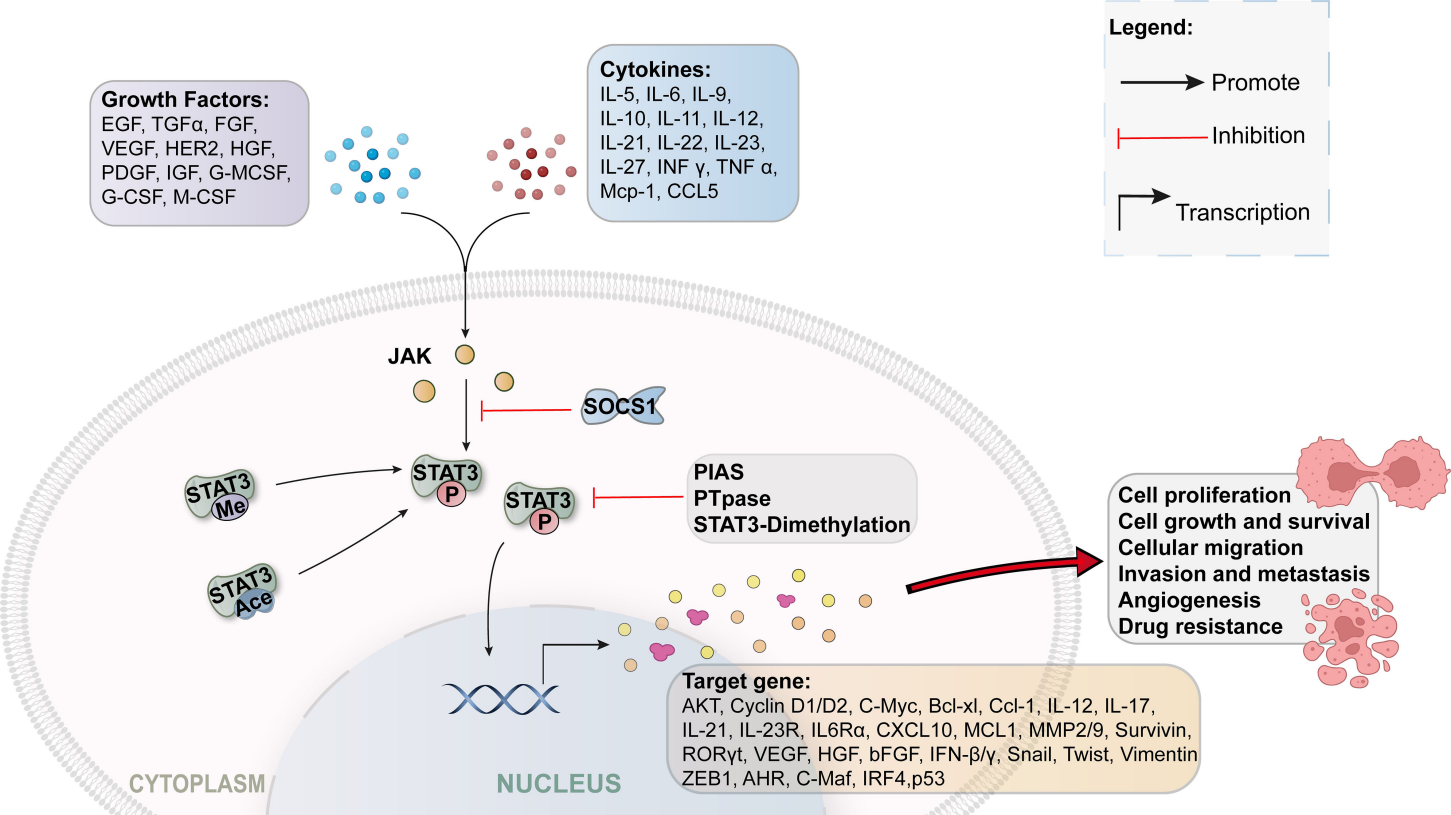
STAT3 signaling pathway.

Wegenka et al. discovered signal transducer and activator of transcription 3 (STAT3) in 1993 (11), an oncogenic transcription factor that mediates cell responses to various growth factors and cytokines (12). STAT proteins include STAT1, STAT2, STAT3, STAT4, STAT5a, STAT5b, and STAT6 (13, 14). The structures of these proteins are similar (12). STAT3 is overexpressed in approximately 70% of cancers and is a potential cytoplasmic protein (15). It is typically located in the cytoplasm of quiescent cells and is usually inactive (16). Once activated, it induces phosphorylation, homodimerization, nuclear translocation, and DNA binding. These enhance tumor proliferation, differentiation, apoptosis, cell transformation, invasion, and angiogenesis (17). Further, STAT3 phosphorylation is significantly associated with increased tumor progression (18). Recently, STAT3 has gained significant attention for its transcriptional activity and role in inflammation, tumor progression, ischemia-reperfusion injury, and stem cell self-renewal (**Figure 1**) (19–21).

STAT3, as an oncogene, is involved in many cellular processes, including invasion, metastasis, angiogenesis, proliferation, and immune escape (22). Increased STAT3 activation has also been identified to perform a role in the progression and metastasis of NPC and is clinically associated with advanced NPC (stage III or IV) (23, 24). An increase in the expression levels of activated STAT3 is strongly linked with a poor prognosis in NPC patients (22).

Aberrant activation and inhibition of STAT3 in some malignant cell lines have been thoroughly examined, rendering it a promising target for the development of anticancer therapies (25, 26). However, many patients with cancer have developed resistance to drugs, making it challenging for typical drug-like compounds to function efficiently (27). Therefore, exploring novel and safer regimens such as STAT3-related targets is important for effective management of patients with NPC.

In this review, we illustrate the close association between STAT3 and NPC progression in order to verify the importance of STAT3-related therapeutic targets.

## 2 Growth factors and cytokines

Cytokine activation of STAT3 is considered the principal pathway in the activation of cancer, and IL-6 is considered an upstream activator of JAK2/STAT3. Zhuang et al. obtained normal nasopharyngeal epithelial tissues and NPC tissues from 117 patients with NPC (28). The serum expression levels of IL-6 and tissue expression levels of p-JAK2, JAK2, p-STAT3, and STAT3 were then measured. Poor TNM stages and lymph node metastases were associated with higher levels of IL-6, p-JAK2, JAK2, p-STAT3, and STAT3 in patients with NPC. Moreover, poor survival rates were observed in patients exhibiting a positive expression of IL-6, p-JAK2, JAK2, p-STAT3, and STAT3. Thus, p-STAT3 and IL-6 are risk factors for poor prognosis in patients with NPC. Recombinant human IL-6 induced cell migration, invasion, and proliferation in SUNE1 and CNE-1 cell lines. AG 490, an inhibitor of IL-6, JAK2, and STAT3, mitigates cell invasion, proliferation, and migration. Therefore, IL-6 enhances tumor cell proliferation, migration, and invasion *via* the JAK2/STAT3 signaling pathway.

### 2.1 The role of STAT3 in NPC pathogenesis

In this paper, we illustrate findings on STAT3 proliferation, migration, anoikis resistance, radiation sensitivity, drug resistance, and the tumor microenvironment in NPC cells. The downstream target genes include cyclin D1, c-Myc, Bcl-xl, Ccl-1, IL-17, IL-21, IL-23R, survivin, RORγt, VEGF, Snail, Twist, ZEB1, AHR, C-Maf, IRF4, IL6Rα, and MMP2/9 (13). STAT3 is an oncogenic transcription factor that enhances cancer cell growth and invasion. Studies conducted to date have demonstrated that activation of STAT3 causes proliferation, invasion, and apoptosis in the pathogenesis of NPC. However, further research is required to clarify the oncogenic role of STAT3 in the pathogenesis of NPC, in addition to the mechanism of action of STAT3-related drugs.

#### 2.1.1 Proliferation and invasion

According to a study by Zhang et al., DANCR can successfully stimulate STAT3 expression in NPC, thereby increasing proliferation and invasion (29). However, DANCR knockdown significantly decreased STAT3 and JAK1 interactions in IL-6-induced NPC, thereby inhibiting NPC cell proliferation and invasion. Another study reported that suppression of miRNA-19b effectively inhibited STAT3 phosphorylation at Tyr705 and Ser727, thereby inhibiting the STAT3 pathway and mitigating the proliferation and invasion of NPC cells. However, STAT3 suppression enhances NPC cell apoptosis by inhibiting the expression of downstream transcriptional targets of the STAT3 signaling pathway, such as anti-apoptotic markers (Mcl-2 and Bcl-2) and proliferative markers (cell cycle protein D1). Cyclic AMP-responsive element-binding protein 1 (CREB1) targeted the secretory granule proteoglycan core protein (SRGN) promoter region to increase SRGN expression in NPC (30). Forkhead box O1 (FoxO1) silencing enhances NPC progression, whereas STAT3 silencing prevents tumor progression. Moreover, the STAT3 silencing-induced downregulation of CREB1 and SRGN was reversed by FoxO1 knockdown. Based on these findings, the FoxO1-miR-148a-5p-CREB1 axis is involved in STAT3-mediated regulation of SRGN and the promotion of nasopharyngeal cancer growth and metastasis, thereby confirming the role of STAT3 in the proliferation, invasion, and anti-apoptotic effects of NPC cells.

#### 2.1.2 Anoikis resistance

Resistance to anoikis is a primary characteristic of tumor cells that metastasize, and STAT3 is closely associated with anoikis resistance in NPC (31). STAT3 expression is reportedly significantly higher in anoikis-resistant NPC cells. Furthermore, anoikis was significantly enhanced with STAT3 inhibitors or STAT3 silencing in anoikis-resistant NPC cells. STAT3 suppression also reduced the resistance of NPC to anoikis and reversed its invasive characteristics. In a recent study, epigallocatechin gallate inhibited the resistance of NPC cells to migration by upregulating miR-296 and targeting STAT3 downregulation (32). This further illustrates the role of STAT3 in anoikis-resistant NPC cell response to other medications or genes.

#### 2.1.3 Radioresistance

STAT3 is also considered to be associated with resistance to radiotherapy in NPC. Du et al. isolated radiation-resistant HK-1R and C666-IR cells from NPC cell lines HK-1 and C666-1 (33). According to miRNA microarray tests, the expression of miR-138-1-3p was highly downregulated in C666-1R cells. A similar significant downregulation was observed using qRT-PCR in HK-1R and C666-IR cells. Following miR-138-1-3p transfection studies, the inhibition of miR-138-1-3p was observed to increase radiation resistance in HK-1 cells, whereas miR-138-1-3p overexpression increased the radiosensitivity of HK-1R cells. In a subsequent experiment, upregulating the expression of miR-138-1-3p decreased the levels of STAT3, JAK2, p-STAT3, p-JAK2, and CRIPTO. This demonstrated that miR-138-1-3p increases the radiosensitivity of NPC by targeting CRIPTO and inhibiting the JAK2/STAT3 signaling pathway. Another study reported that creatine kinase, mitochondrial 1 (CKMT1)-overexpressing CNE-1 cells reduced cell cycle arrest at the G2/M phase, experienced an increased cell colony formation rate, decreased apoptosis rate, increased STAT3 phosphorylation levels after radiotherapy, and decreased cleaved poly ADP-ribose polymerase (c-PARP) levels (34). In contrast, CKMT1 knockdown in CNE-2 cells reduced the clone formation rate, increased cell cycle arrest at the G2/M phase, and apoptosis rate, and decreased STAT3 phosphorylation levels, and c-PARP levels. These results demonstrated that greater CKMT1 levels decreased radiosensitivity in NPC cells through increasing STAT3 phosphorylation.

#### 2.1.4 Chemotherapy resistance

STAT3 is also involved in NPC chemoresistance. Gao et al. demonstrated that the expression of proliferating cell nuclear antigen (PCNA), p-STAT3, and STAT3 was significantly higher in CNE1/Taxol drug-resistant cells than in CNE1 cells; however, the expression of miR-29a and apoptosis were significantly decreased (35). The expression levels of PCNA and p-STAT3 were dramatically elevated. The expression of PCNA, p-STAT3 and STAT3 were reduced by si-STAT3 and miR-29a transfection, which also inhibited proliferation and enhanced apoptosis. This demonstrates that miR-29a downregulation is associated with drug resistance in the NPC cell line CNE-1 and that miR-29a upregulation reduces the resistance of CNE-1 cells to Taxol by inhibiting Bcl-2 and STAT3 expression. Another study also demonstrated that AGK levels were elevated in Taxol-resistant NPC cells and that AGK knockdown inhibited Taxol resistance in CNE2-TR and CNE1-TR cells (36). Through regulating the JAK2/STAT3/FOXM1 axis, *in vivo* tumor xenograft assays further demonstrated that knockdown of AGK inhibited tumor development and decreased resistance to paclitaxel. Zeng et al. also observed that mesenchymal stem cells (MSCs) secreted IL-6 in the TME, leading to STAT3 activation in cancer cells, thereby accelerating the oncogenesis of NPC and their resistance to cisplatin therapy (37). However, in these two studies, only the mechanism by which STAT3 and its associated genes affected NPC resistance to chemotherapeutic drugs were investigated and their specific applications were not examined. Zhu et al. demonstrated that NPC cells overcame cisplatin resistance after treatment with stattic (a STAT3 inhibitor) (38). They discovered that cisplatin exhibited a synergistic effect on resistant cells with both JAK2 (LY2784544) and STAT3 inhibitors. However, further clinical studies on STAT3 inhibitors in NPC chemoresistance are highly recommended.

### 2.2 STAT3 as a therapeutic target in NPC

STAT3 is highly expressed in NPC. It enhances the proliferation and migration of NPC, and its inhibition significantly slows the progression of NPC. To prevent the spread of NPC and enhance the effectiveness of radiotherapy, suppressing STAT3 is a valuable therapeutic option. Inhibiting the upstream pathways associated with NPC (such as JAK, IL-6, and SRC) inhibits STAT3 action. For example, JAK inhibitors (ruxolitinib, baricitinib, tofacitinib, fedratinib, momelotinib, pacritinib, NDI-031301, and LY2784544) have been used to treat myeloproliferative tumors, gastric adenocarcinoma, prostate cancer, and other cancers (39). Some studies revealed that JAK inhibitors (LY2784544) and STAT3 inhibitors mitigate the resistance of NPC cells to cisplatin. However, the effectiveness of JAK inhibitors in treating NPC has not been studied. Among the IL-6 inhibitors (sirukumab, siltuximab, clazakizumab, olokizumab, and MEDI5117); the most effective is siltuximab, a hybrid mouse-human antibody (40). Siltuximab has been shown to exert antitumor effects on preclinical models of solid tumors (e.g., lung, prostate, and ovarian cancers). This effect is currently limited, and the use of IL-6 inhibitors to treat NPC requires the development of other combination therapies.

However, STAT3 can be directly used as a target for gene therapy. STAT3 was presumed to be uninhibited because it lacks enzymatic activity. However, its significant role in accelerating the evolution of tumors has led to the development of drugs that can suppress its function and expression.

Regulators of STAT3 modulate host immunity to cancer through direct inhibition of STAT3 expression/inhibition of the STAT3 upstream pathway. However, some immune cells (e.g., natural killer cells, macrophages, and Th1 cells) directly or indirectly (through the release of cytokines) destroy tumor cells (41). Tumor-associated macrophages and myeloid-derived suppressor cells (MDSCs) enhance cancer progression by secretion of angiogenic, metastatic, and growth factors (42). Current research has revealed that STAT3 is involved in the immune escape of tumor cells as it suppresses anticancer immune cells and activates pro-cancer immune cells. Similarly for NPC, we determined that STING suppressed the induction of NPC-derived MDSCs by increasing the expression of SOCS1 in tumor cells and MDSCs (43). Through its structural domain, SH2, SOCS1 physically interacts with STAT3, preventing its phosphorylation and dimerization. Moreover, SOCS1 inhibits MDSC induction by inhibiting the synthesis of GM-CSF and IL-6. This suggests that STING may target STAT3 to inhibit the production of GM-CSF and IL-6, thereby inhibiting EBV-associated NPC progression.

These drugs are currently undergoing clinical trials. The first direct inhibitors of STAT3 were developed based on peptidomimetics (PM-73G, ISS-610) and tyrosine-phosphorylated peptides (PY*LKTK) that bind to the SH2 domain of STAT3 and disrupt its DNA-binding activity and dimerization (40). The growth of tumors with increased levels of activated STAT3 are inhibited by non-peptide SH2 structural domain inhibitors such as stattic, LLL-3, STA-21, BP-1-102, STX-0119, HJC0123, S3I-201, and WP1066. In preclinical models, HJC0123, STX-0119, and BP-1-102 are orally bioavailable. The non-peptide STAT3-SH2 structural domain antagonists, C188-9, OPB-51602, and OPB-31121 are currently in the early phase of clinical trials. The phase 0 clinical trial for STAT3 decoy oligonucleotide against HNSCC (NCT00696176) is complete. Phase I/II clinical trials for azd9150 against metastatic HNSCC have been concluded (NCT02499328). This review illustrates the mechanisms by which precise targeting of STAT3 can be applied to treat head and neck tumors. In conclusion, the development of highly specific and selective STAT3 inhibitors offers a key prospect for future therapeutic and clinical trials in tumors, including NPC.

## 3 Phytochemicals

NPC is frequently treated with radiotherapy as the first-line treatment; however, NPC resistance to radiotherapy reduces clinical treatment efficacy. Therefore, drugs that inhibit the progression of NPC are of great interest to clinicians. There is an urgent need for new therapeutic agents that can treat NPC patients with minimal side effects and a low risk of recurrence. Natural products provide a rich bioactive structural base for the development of novel drugs. Some phytochemical factors affect internal signaling pathways, including STAT3, nuclear factor-kappa B (NF-κB), extracellular signal-regulated kinase (ERK)-1/2, and phosphatidylinositol 3−kinase (PI3K)/protein kinase B (AKT) (44, 45). Some natural products reportedly affect NPC progression of NPC by inhibiting STAT3 activity, including apigenin, garcinone C, β-elemene, caffeic acid phenethyl ester (CAPE), brevilin A, limonin, curcumin, wikstroflavone B, and arnicolide D.

### 3.1 Apigenin decreases STAT3 activity and inhibits the proliferation of NPC cells

Apigenin is a natural plant flavonoid (4, 5, 7-trihydroxyflavone) widely found in fruits and vegetables. It has anti-proliferative, anti-inflammatory, and anti-angiogenic effects in tumors and metabolic diseases (46). It also exhibits anti-proliferative effects on cancers, including colorectal and ovarian cancers (47–49).

Previous studies demonstrated that complement 5a (C5a) can induce the proliferation of NPC cells through promote the expression of P300/CBP-related factor (PCAF) and PCAF-mediated STAT3 acetylation (50). Recently, studies discovered that apigenin lowered the expression of C5aR, as well as the proliferation of NPC cells *in vitro*. Apigenin also decreases STAT3 activity and inhibits the C5aR/PCAF/STAT3 axis-mediated proliferation of NPC cells induced through C5a. Interestingly, the combination of apigenin and cetuximab was shown to better inhibit the viability and suppress the growth of NPC cell lines (CNE2 and HONE1) compared to apigenin or cetuximab alone (51). In addition, this drug combination produced a greater pro-apoptotic effect and inhibition effect of p-STAT3 (52). Cetuximab has become a key chemotherapy combination for patients with NPC, especially recurrent and/or metastatic NPC (53). These results suggest that the combination of apigenin and cetuximab may be more effective in addressing the wide variation in individual tolerance and toxicity to chemotherapy.

### 3.2 Garcinone C exerts antitumor activity *via* regulating ATR/Stat3/4E−BP1

Garcinone C is a compound isolated from Garcinia oblongifolia Champ. It possesses cytotoxic effects in some cancers, such as colon cancer and NPC (54). Moreover, it significantly inhibits the expression of STAT3, thereby modulating the viability of the human NPC cell lines HONE1, HK1, CNE1, and CNE2 (55). After a high-dose (10 M) treatment with garcinone C, several cell lines demonstrated necrotic morphological changes such as endoplasmic reticulum expansion, rough endoplasmic reticulum degranulation, cell swelling, vacuolar degeneration, and mitochondrial swelling. Garcinone C is a potential drug for the treatment of NPC based on its ability to reduce NPC growth *in vitro*. Due to the limitations of the current study, *in vivo* experiments and clinical trials are both still limited. Further in-depth investigation of the carcinogenic effect of garcinone C in clinical use for NPC is required.

### 3.3 β-elemene inhibited NPC cell proliferation by suppressing STAT3 expression

β-elemene is derived from the traditional Chinese medicinal herb, Zedoary, which has demonstrated antitumor effects in various cancers (56, 57). The China Food and Drug Administration (CFDA) approved Elemene injection (CFDA No. H10960114) and Elemene oral emulsion (CFDA No. H20010338) as broad-spectrum antitumor agents to treat NPC, brain cancer, liver cancer, and lung cancer in 1994 (58). By inhibiting STAT3 expression, Wu et al. revealed that β-elemene inhibited NPC cell proliferation and decreased enhancer of zeste homolog 2 (EZH2) and DNA methyltransferase 1 (DNMT1) levels (59). The total reaction of β-elemene is influenced by interactions between DNMT1 and EZH2 as well as between Stat3, EZH2, and DNMT1. In the current study, we identified the restrictive effect of β-elemene on NPC development. However, its low bioavailability and lipophilicity limit its clinical application. More effective structural modifications of β-elemene are needed to obtain effective drugs for clinical application.

### 3.4 Caffeic acid phenethyl ester (CAPE) mitigated the proliferation and invasion of NPC by inhibiting STAT3 phosphorylation through the MAPK pathway

CAPE is a bioactive ingredient extracted from propolis known for its anticancer properties. Studies have shown that CAPE acts as an effective sensitizer for radiotherapy, including NPC (60). Chiang et al. determined that it mitigated the proliferation and invasion of NPC by upregulating N-myc downstream-regulated gene 1 (NDRG1) expression. Immunoblot assays revealed that STAT3 phosphorylation was inhibited as a time-dependent manner after CAPE treatment (30 µM). Meanwhile, results of reporter assays using the STAT3-specific reporter vector involving the STAT3 binding site showed that CAPE downregulated the activity of STAT3. CAPE treatments inhibit STAT3 phosphorylation and reduce STAT3 activity (61). CAPE may be a potential STAT3 inhibitor, however, its specific mechanism and *in vitro* experiments require further investigation.

### 3.5 Brevilin A has anticancer effects on NPC *via* blocking the STAT3 signaling pathway

The sesquiterpene lactone brevilin A is extracted from Centipeda minima (CM) (62). Brevilin A has anticancer effects on multiple myeloma, as well as breast, colorectal, and lung cancers (63–67). It also inhibits the growth of NPC cells, and its efficacy is comparable to cisplatin *in vivo* without its associated toxicity by inhibiting the STAT3 and PI3K/AKT/mTOR signaling pathways (68–70) and activating the caspase signaling pathway. In the *in vitro* experiments, cisplatin-treated mice exhibited a significant decrease in body weight, however, brevilin A-treated mice showed no significant change, indicating that brevilin A had a relative lack of toxicity. These results offer a framework for the preclinical development of brevilin A as a therapeutic agent with an NPC chemotherapeutic drug.

### 3.6 Limonin mitigated the stemness of NPC cells by inhibiting STAT3 transcriptional activity

Limonin, a triterpenoid agent, is a critical secondary metabolite with high bioactivity (71). Recent studies have shown that it has antitumor, neuroprotective, antioxidant, and other biological properties (72, 73). Based on RNA-sequencing analysis, the mechanistic studies showed that limonin suppressed Stat3-driven gene transcription. The inhibitory effects of limonin were reversed by an activator of Stat3 (IL-6 or Colivelin). This research proved that limonin mitigates the stemness of NPC cells by inhibiting STAT3 transcriptional activity, thereby reducing radiation sensitivity (74). NPC is highly sensitive to radiotherapy (75); thus, radiotherapy has been the first choice of treatment for NPC (76). However, resistance to radiotherapy has prompted the search for novel treatment methods. Therefore, inhibiting the STAT3 signaling pathway to improve radiotherapy sensitivity may offer high value to patients resistant to radiotherapy.

### 3.7 Curcumin and wikstroflavone B suppressed the proliferation and metastasis of NPC *via* restraining the FAK/STAT3 signaling pathway

Curcumin, a polyphenol purified from Zingiberaceae, expresses potent biological activity against various cancer types (77). In previous studies, curcumin was shown to be effective at inhibiting the progression and promoting the sensitivity of NPC to radiotherapy (78, 79). Furthermore, a recent study by Shao et al. determined that a new biflavonoid compound (Wikstroflavone B, WFB) isolated from Wikstroemia indica could synergistically inhibit the effects of curcumin on the metastasis and proliferation of NPC, providing a rational approach for the development of anticancer drugs (80). CUR/WFB treatment blocked the phosphorylation of STAT3 and FAK in NPC cells. The combination of WFB and curcumin can enhance the inhibition of survival and metastasis of NPC cells, and improve the efficacy and reduce the side effects of each drug.

### 3.8 Arnicolide D inhibits NPC cell viability *via* inhibiting the STAT3 signaling pathway

Arnicolide D is a sesquiterpene lactone extracted from CM. CM extracts have been used to relieve pain, reduce swelling, and treat rhinitis. Recent studies have reported that CM extracts have antitumor effects against colorectal carcinoma, breast cancer, and NPC (81). Arnicolide D, acting on HONE1, C666-1, CNE-1, CNE-2, and SUNE-1, inhibits NPC cell viability in a concentration- and time-dependent manner. The molecular mechanisms of apoptosis induction and cell cycle regulation revealed that arnicolide D activated the caspase signaling pathway and inhibited the STAT3 and PI3K/AKT/mTOR signaling pathways to promote cell apoptosis and induce cell cycle arrest at the G2/M phase (**Table 1**) (82).

**Table 1:** STAT3target genes/protein/drugs in NPC.

		STAT3 target genes/protein/drugs.			
STAT3-regulated genes/protein/drug	Mechanism	Cell line	Radiation	Effect	Reference
AG 490	inhibited IL-6 and JAK2/STAT3	CNE-1 and SUNE1 cell lines	NO	inhibited cell proliferation, invasion and migration	(28)
STING	target STAT3 to inhibit the production of GM-CSF and IL-6	CNE2, C666, and TW03 cell lines	NO	inhibited EBV-associated NPC progression	(43)
DANCR	interacted with STAT3 and enhanced JAK1 binding to STAT3 to strengthen IL-6/JAK1/STAT3 signaling	CNE1, CNE2, HNE1, HNE2, HONE1, 5–8 F and 6-10B cell lines	NO	promoted NPC cell proliferation, migration and invasion	(29)
FoxO1	target STAT3 to improve the expression of CREB1 and SRGN	HONE1, 5-8F cell lines	NO	promoted the growth and metastasis of NPC	(30)
Stattic	inhibited the expression of STAT3	TW01 and TW06	NO	promoted the ability of anoikis-resistant and promoted the cisplatin resistance	(38)
miR-296	downregulated the STAT3 expression	anoikis-resistant NPC cells	NO	inhibited the migratory properties	(32)
miR-138-1-3p	inhibited EMT and targeting CRIPTO to reduce the activation of the JAK2/STAT3 pathway	C666-IR and HK-1R NPC cell lines	YES	reversed the radioresistant characteristics of NPC stem cells	(33)
CKMT1	exhibited radiosensitivity	CNE-1 and CNE-2 cell lines	YES	promoted STAT3 phosphorylation	(34)
miR-29a	inhibited STAT3 and Bcl-2 expression	CNE-1 cell lines	NO	reduced the resistance of NPC cells to Taxol	(35)
AGK	regulated the JAK2/ STAT3/ FOXM1 axis	CNE-1 and CNE-2 cell lines		promoted the resistance of NPC cells to Taxol	(36)
LY2784544	inhibited the expression of JAK			reduced the resistance of NPC cells to cisplatin	(38)
apigenin	deactivated C5aR/PCAF/STAT3 axis	C666-1 cell lines	NO	inhibited proliferation of NPC cells induced by C5a	(50)
garcinone C	inhibited the expression of Stat3	CNE1, CNE2, HK1 and HONE1 cell lines	NO	inhibited the cell proliferation and colony-formation ability of NPC cells *in vitro*	(55)
β-elemene	reduced phosphorylation of Stat3	C666-1 and HNE2 cell lines	NO	inhibited growth and induced cell cycle arrest in NPC cells	(59)
CAPE	inhibited phosphorylation of STAT3	TW01 and TW04 cell lines	NO	attenuated NPC cell proliferation and invasion	(61)
brevilin A	inhibited PI3K/AKT/mTOR and STAT3 signaling pathways	CNE-1, CNE-2, SUNE-1, HONE1, and C666-1 cell lines	NO	inhibited cell proliferation and induced apoptosis	(70)
limonin	suppressed Stat3 transcriptional activity	FaDu and SCC25 cell lines	YES	reduce the stemness and radiosensitivity of NPC cells	(74)
wikstroflavone B	inhibit the effect of curcumin	CNE1, CNE2, HONE1 and C666-1	NO	inhibited cell proliferation and metastasis	(80)
arnicolide D	inhibit the PI3K/AKT/mTOR and STAT3 signaling pathways	CNE-1, CNE-2, SUNE-1, HONE1 and C666-1	NO	inhibit NPC cell viability, induce cell cycle arrest at G2/M, and induce cell apoptosis	(82)

## 4 Conclusion

NCP has a high tendency for local invasion of lymph nodes and distant metastasis. Recurrence and metastasis have been challenging for treating NPC. Due to its function in various biological processes and disorders, including cancer, the transcription factor STAT3 has been extensively studied for over two decades. STAT3 is engaged in a wide range of cellular functions, and its overactivation is associated with the onset of many tumors. Therefore, it is a critical target for the development of novel drugs due to the limited efficacy of traditional drug-like compounds. This limited efficacy is attributed to the high drug resistance rate among patients with cancer. Identifying new targets related to STAT3 will advance the treatment of NPC and improve the prognosis of patients as STAT3 activation is involved in the progression and metastasis of NPC.

## Author contributions

YS wrote the first draft of the manuscript. JX and LM wrote sections of the manuscript. YW prepared figure and table. JQ revised the manuscript. All authors contributed to manuscript revision, read, and approved the submitted version.

## Conflict of interest

The authors declare that the research was conducted in the absence of any commercial or financial relationships that could be construed as a potential conflict of interest.

## Publisher’s note

All claims expressed in this article are solely those of the authors and do not necessarily represent those of their affiliated organizations, or those of the publisher, the editors and the reviewers. Any product that may be evaluated in this article, or claim that may be made by its manufacturer, is not guaranteed or endorsed by the publisher.

## References

[1] Arshad S Naveed M Ullia M Javed K Butt A Khawar M. Targeting STAT-3 signaling pathway in cancer for development of novel drugs: Advancements and challenges. Genet Mol Biol (2020) 43:e20180160. doi: 10.1590/1678-4685-gmb-2018-0160 32167126 PMC7198026

[2] Chen Y-P Chan ATC Le Q-T Blanchard P Sun Y Ma J. Nasopharyngeal carcinoma. Lancet (2019) 394:64–80. doi: 10.1016/S0140-6736(19)30956-0 31178151

[3] Lo KW To KF Huang DP. Focus on nasopharyngeal carcinoma. Cancer Cell (2004) 5:423–8. doi: 10.1016/S1535-6108(04)00119-9 15144950

[4] Liu SL Bian LJ Liu ZX Chen QY Sun XS Sun R. Development and validation of the immune signature to predict distant metastasis in patients with nasopharyngeal carcinoma. J Immunother Cancer (2020) 8:e000205. doi: 10.1136/jitc-2019-000205 32303611 PMC7204817

[5] Lee AWM Ng WT Chan JYW Corry J Makitie A Mendenhall WM. Management of locally recurrent nasopharyngeal carcinoma. Cancer Treat Rev (2019) 79:101890. doi: 10.1016/j.ctrv.2019.101890 31470314

[6] Stelow E Wenig BJH Pathology N. Update from the 4th edition of the world health organization classification of head and neck tumours: Nasopharynx. Head Neck Pathol (2017) 11:16–22. doi: 10.1007/s12105-017-0787-0 28247232 PMC5340728

[7] Young L Dawson C. Epstein-Barr Virus and nasopharyngeal carcinoma. Chin J Cancer (2014) 33:581–90. doi: 10.5732/cjc.014.10197 PMC4308653 25418193

[8] Nagaprashantha L Vatsyayan R Lelsani P Awasthi S Singhal S. The sensors and regulators of cell-matrix surveillance in anoikis resistance of tumors. Int J Cancer (2011) 128:743–52. doi: 10.1002/ijc.25725 PMC3292620 20949625

[9] Campion N Ally M Jank B Ahmed J Alusi G. The molecular march of primary and recurrent nasopharyngeal carcinoma. Oncogene (2021) 40:1757–74. doi: 10.1038/s41388-020-01631-2 33479496

[10] Morimoto-Tomita M Ohashi Y Matsubara A Tsuiji M Irimura TJC. Mouse colon carcinoma cells established for high incidence of experimental hepatic metastasis exhibit accelerated and anchorage-independent growth. Clin Exp Metastasis (2005) 22:513–21. doi: 10.1007/s10585-005-3585-0 16320114

[11] Wegenka U Lütticken C Buschmann J Yuan J Lottspeich F Müller-Esterl W. The interleukin-6-activated acute-phase response factor is antigenically and functionally related to members of the signal transducer and activator of transcription (STAT) family. Mol Cell Biol (1994) 14:3186–96. doi: 10.1128/mcb.14.5.3186-3196.1994 PMC358686 8164674

[12] Gharibi T Babaloo Z Hosseini A Abdollahpour-Alitappeh M Hashemi V Marofi F. Targeting STAT3 in cancer and autoimmune diseases. Eur J Pharmacol (2020) 878:173107. doi: 10.1016/j.ejphar.2020.173107 32278856

[13] Zou S Tong Q Liu B Huang W Tian Y Fu X. Targeting STAT3 in cancer immunotherapy. Mol Cancer (2020) 19:145. doi: 10.1186/s12943-020-01258-7 32972405 PMC7513516

[14] Verhoeven Y Tilborghs S Jacobs J De Waele J Quatannens D Deben C. The potential and controversy of targeting STAT family members in cancer. Semin Cancer Biol (2020) 60:41–56. doi: 10.1016/j.semcancer.2019.10.002 31605750

[15] Wei N Li J Fang C Chang J Xirou V Syrigos N. Targeting colon cancer with the novel STAT3 inhibitor bruceantinol. Oncogene (2019) 38:1676–87. doi: 10.1038/s41388-018-0547-y 30348989

[16] Mohassab A Hassan H Abdelhamid D Abdel-Aziz M. STAT3 transcription factor as target for anti-cancer therapy. Pharmacol Reports: PR (2020) 72:1101–24. doi: 10.1007/s43440-020-00156-5 32880101

[17] Jaskiewicz A Domoradzki T Pajak B. Targeting the JAK2/STAT3 pathway-can we compare it to the two faces of the God janus? Int J Mol Sci (2020) 21:8261. doi: 10.3390/ijms21218261 PMC7663396 33158194

[18] Gu Y Mohammad I Liu Z. Overview of the STAT-3 signaling pathway in cancer and the development of specific inhibitors. Oncol Lett (2020) 19:2585–94. doi: 10.3892/ol.2020.11394 PMC7068531 32218808

[19] Liang Y Kong D Zhang Y Li S Li Y Ramamoorthy A. Fisetin inhibits cell proliferation and induces apoptosis *via* JAK/STAT3 signaling pathways in human thyroid TPC 1 cancer cells. Biotechnol Bioprocess Engineering (2020) 25:197–205. doi: 10.1007/s12257-019-0326-9

[20] Liang T He Y Chang Y Liu X. 6-shogaol a active component from ginger inhibits cell proliferation and induces apoptosis through inhibition of STAT-3 translocation in ovarian cancer cell lines (A2780). Biotechnol Bioprocess Engineering (2019) 24:560–7. doi: 10.1007/s12257-018-0502-3

[21] Debnath B Xu S Neamati N. Small molecule inhibitors of signal transducer and activator of transcription 3 (Stat3) protein. J Med Chem (2012) 55:6645–68. doi: 10.1021/jm300207s 22650325

[22] Lee H Jeong AJ Ye S-K. Highlighted STAT3 as a potential drug target for cancer therapy. BMB Rep (2019) 52:415–23. doi: 10.5483/BMBRep.2019.52.7.152 PMC6675244 31186087

[23] Liu Y Tan Y Wang Z Zeng L Lu Z Li L. Phosphorylation and nuclear translocation of STAT3 regulated by the Epstein-Barr virus latent membrane protein 1 in nasopharyngeal carcinoma. Int J Mol Med (2008) 21:153–62. doi: 10.3892/ijmm.21.2.153 18204781

[24] Wang Z Luo F Li L Yang L Hu D Ma X. STAT3 activation induced by Epstein-Barr virus latent membrane protein1 causes vascular endothelial growth factor expression and cellular invasiveness *via* JAK3 and ERK signaling. Eur J Cancer (2010) 46:2996–3006. doi: 10.1016/j.ejca.2010.07.008 20709526

[25] Busker S Qian W Haraldsson M Espinosa B Johansson L Attarha S. Irreversible TrxR1 inhibitors block STAT3 activity and induce cancer cell death. Sci Adv (2020) 6:eaax7945. doi: 10.1126/sciadv.aax7945 32219156 PMC7083616

[26] Im J Kim B Lee K Chun S Kang M Won MJO. DDIAS promotes STAT3 activation by preventing STAT3 recruitment to PTPRM in lung cancer cells. Oncogenesis (2020) 9:1. doi: 10.1038/s41389-019-0187-2 31900385 PMC6949220

[27] Sarwar M Xia Y Liang Z Tsang S Zhang H. Mechanistic pathways and molecular targets of plant-derived anticancer -kaurane diterpenes. Biomolecules (2020) 10:144. doi: 10.3390/biom10010144 PMC7023344 31963204

[28] Zhuang M Ding X Song W Chen H Guan H Yu Y. Correlation of IL-6 and JAK2/STAT3 signaling pathway with prognosis of nasopharyngeal carcinoma patients. Aging (2021) 13:16667–83. doi: 10.18632/aging.203186 PMC8266356 34165442

[29] Zhang X Yang J Bian Z Shi D Cao Z. Long noncoding RNA DANCR promotes nasopharyngeal carcinoma progression by interacting with STAT3, enhancing IL-6/JAK1/STAT3 signaling. Biomed pharmacother = Biomed Pharmacother (2019) 113:108713. doi: 10.1016/j.biopha.2019.108713 30849642

[30] Wang YL Ren D Lu JL Jiang H Wei JZ Lan J. STAT3 regulates SRGN and promotes metastasis of nasopharyngeal carcinoma through the FoxO1-miR-148a-5p-CREB1 axis. Lab Invest (2022) 102:919–34. doi: 10.1038/s41374-022-00733-7 35562411

[31] Wang LN Zhang ZT Wang L Wei HX Zhang T Zhang LM. TGF-beta1/SH2B3 axis regulates anoikis resistance and EMT of lung cancer cells by modulating JAK2/STAT3 and SHP2/Grb2 signaling pathways. Cell Death Dis (2022) 13:472. doi: 10.1038/s41419-022-04890-x 35589677 PMC9120066

[32] Lin C Wang H Chen T Chiang M Hung P Chen Y. Involvement of MicroRNA-296 in the inhibitory effect of epigallocatechin gallate against the migratory properties of anoikis-resistant nasopharyngeal carcinoma cells. Cancers (2020) 12:15. doi: 10.3390/cancers12040973 PMC7226234 32326395

[33] Du T Jiang J Chen Y Zhang N Chen G Wang X. MiR-138-1-3p alters the stemness and radiosensitivity of tumor cells by targeting CRIPTO and the JAK2/STAT3 pathway in nasopharyngeal carcinoma. Ann Trans Med (2021) 9:485. doi: 10.21037/atm-21-521 PMC8039661 33850882

[34] Lan R Huang F Zhong G Chen R Wang Z Chen J. Effects of CKMT1 on radiosensitivity of nasopharyngeal carcinoma cells. Int J Radiat Biol (2019) 95:597–606. doi: 10.1080/09553002.2019.1554919 30507333

[35] Gao J Shao Z Yan M Fu T Zhang L Yan Y. Targeted regulationof STAT3 by miR-29a in mediating taxol resistance of nasopharyngeal carcinoma cell line CNE-1. Cancer Biomark: Section A Dis Markers (2018) 22:641–8. doi: 10.3233/CBM-170964 PMC13078501 29914005

[36] Zhao C Chen H Zhao F Feng H Su J. Acylglycerol kinase promotes paclitaxel resistance in nasopharyngeal carcinoma cells by regulating FOXM1 *via* the JAK2/STAT3 pathway. Cytokine (2021) 148:155595. doi: 10.1016/j.cyto.2021.155595 34116927

[37] Zeng J Chen S Li C Ye Z Lin B Liang Y. Mesenchymal stem/stromal cells-derived IL-6 promotes nasopharyngeal carcinoma growth and resistance to cisplatin *via* upregulating CD73 expression. J Cancer (2020) 11:2068–79. doi: 10.7150/jca.37932 PMC7052921 32127934

[38] Zhu X Liu L Wang Y Cong J Lin Z Wang Y. LncRNA MIAT/HMGB1 axis is involved in cisplatin resistance regulating IL6-mediated activation of the JAK2/STAT3 pathway in nasopharyngeal carcinoma. Front Oncol (2021) 11:651693. doi: 10.3389/fonc.2021.651693 34094941 PMC8173225

[39] McLornan D Pope J Gotlib J Harrison C. Current and future status of JAK inhibitors. Lancet (London England) (2021) 398:803–16. doi: 10.1016/S0140-6736(21)00438-4 34454676

[40] Johnson D O’Keefe R Grandis J. Targeting the IL-6/JAK/STAT3 signalling axis in cancer. Nat Rev Clin Oncol (2018) 15:234–48. doi: 10.1038/nrclinonc.2018.8 PMC5858971 29405201

[41] Trinchieri G. Interleukin-12 and the regulation of innate resistance and adaptive immunity. Nat Rev Immunol (2003) 3:133–46. doi: 10.1038/nri1001 12563297

[42] Matsukawa A Kudo S Maeda T Numata K Watanabe H Takeda K. Stat3 in resident macrophages as a repressor protein of inflammatory response. J Immunol (2005) 175:3354–9. doi: 10.4049/jimmunol.175.5.3354 16116228

[43] Zhang C Ye S Ni J Cai T Liu Y Huang D. STING signaling remodels the tumor microenvironment by antagonizing myeloid-derived suppressor cell expansion. Cell Death Differ (2019) 26:2314–28. doi: 10.1038/s41418-019-0302-0 PMC6889506 30816302

[44] Liu L Leung K Chan D Wang Y Ma D Leung C. Identification of a natural product-like STAT3 dimerization inhibitor by structure-based virtual screening. Cell Death Disease (2014) 5:e1293. doi: 10.1038/cddis.2014.250 24922077 PMC4611723

[45] Rana P Shrama A Mandal C. Molecular insights into phytochemicals-driven break function in tumor microenvironment. J Food Biochem (2021) 45:e13824. doi: 10.1111/jfbc.13824 34219240

[46] Meresman G Götte M Laschke M. Plants as source of new therapies for endometriosis: a review of preclinical and clinical studies. Hum Reprod uUpdate (2021) 27:367–92. doi: 10.1093/humupd/dmaa039 33124671

[47] Khan H Ullah H Martorell M Valdes S Belwal T Tejada S. Flavonoids nanoparticles in cancer: Treatment, prevention and clinical prospects. Semin Cancer Biol (2021) 69:200–11. doi: 10.1016/j.semcancer.2019.07.023 31374244

[48] Yang C Song J Hwang S Choi J Song G Lim W. Apigenin enhances apoptosis induction by 5-fluorouracil through regulation of thymidylate synthase in colorectal cancer cells. Redox Biol (2021) 47:102144. doi: 10.1016/j.redox.2021.102144 34562873 PMC8476449

[49] Niu N Yao J Bast R Sood A Liu J. IL-6 promotes drug resistance through formation of polyploid giant cancer cells and stromal fibroblast reprogramming. Oncogenesis (2021) 10:65. doi: 10.1038/s41389-021-00349-4 34588424 PMC8481288

[50] Cai K Wan Y Wang Z Wang Y Zhao X Bao X. C5a promotes the proliferation of human nasopharyngeal carcinoma cells through PCAF-mediated STAT3 acetylation. Oncol Rep (2014) 32:2260–6. doi: 10.3892/or.2014.3420 25174320

[51] Zhang Y Cao Y Zhang L Feng C Zhou G Wen G. Apigenin inhibits C5a-induced proliferation of human nasopharyngeal carcinoma cells through down-regulation of C5aR. Biosci Rep (2018) 38:BSR20180456. doi: 10.1042/BSR20180456 29685955 PMC6048209

[52] Hu WJ Liu J Zhong LK Wang J. Apigenin enhances the antitumor effects of cetuximab in nasopharyngeal carcinoma by inhibiting EGFR signaling. BioMed Pharmacother (2018) 102:681–8. doi: 10.1016/j.biopha.2018.03.111 29604587

[53] Ueda Y Enokida T Okano S Fujisawa T Ito K Tahara M. Combination treatment with paclitaxel, carboplatin, and cetuximab (PCE) as first-line treatment in patients with recurrent and/or metastatic nasopharyngeal carcinoma. Front Oncol (2020) 10:571304. doi: 10.3389/fonc.2020.571304 33117701 PMC7575747

[54] Chen J Qiu S Kim JT Cho JS Moon JH Zhou Y. Garcinone c suppresses colon tumorigenesis through the Gli1-dependent hedgehog signaling pathway. Phytomedicine (2020) 79:153334. doi: 10.1016/j.phymed.2020.153334 32920288

[55] Xia Y Liu X Zou C Feng S Guo H Yang Y. Garcinone c exerts antitumor activity by modulating the expression of ATR/Stat3/4E−BP1 in nasopharyngeal carcinoma cells. Oncol Rep (2018) 39:1485–93. doi: 10.3892/or.2018.6218 29344638

[56] Yang D Xu X Wang X Feng W Shen X Zhang J. β-elemene promotes the senescence of glioma cells through regulating YAP-CDK6 signaling. Am J Cancer Res (2021) 11:370–88. PMC7868755 33575077

[57] Song H Liu B Dong B Xu J Zhou H Na S. Exosome-based delivery of natural products in cancer therapy. Front Cell (2021) 9:650426. doi: 10.3389/fcell.2021.650426 PMC7960777 33738290

[58] Bai Z Yao C Zhu J Xie Y Ye XY Bai R. Anti-tumor drug discovery based on natural product beta-elemene: Anti-tumor mechanisms and structural modification. Molecules (2021) 26:1499. doi: 10.3390/molecules26061499 33801899 PMC7998186

[59] Wu J Tang Q Yang L Chen Y Zheng F Hann S. Interplay of DNA methyltransferase 1 and EZH2 through inactivation of Stat3 contributes to β-elemene-inhibited growth of nasopharyngeal carcinoma cells. Sci Rep (2017) 7:509. doi: 10.1038/s41598-017-00626-6 28360411 PMC5428779

[60] Liang Y Feng G Wu L Zhong S Gao X Tong Y. Caffeic acid phenethyl ester suppressed growth and metastasis of nasopharyngeal carcinoma cells by inactivating the NF-kappaB pathway. Drug Des Devel Ther (2019) 13:1335–45. doi: 10.2147/DDDT.S199182 PMC6499142 31118570

[61] Chiang K Yang S Chang K Feng T Chang K Tsui K. Caffeic acid phenethyl ester induces -myc downstream regulated gene 1 to inhibit cell proliferation and invasion of human nasopharyngeal cancer cells. Int J Mol Sci (2018) 19:1397. doi: 10.3390/ijms19051397 PMC5983775 29738439

[62] Qu Z Lin Y Mok D Bian Q Tai W Chen S. Brevilin a, a natural sesquiterpene lactone inhibited the growth of triple-negative breast cancer cells *via* Akt/mTOR and STAT3 signaling pathways. OncoTargets Ther (2020) 13:5363–73. doi: 10.2147/OTT.S256833 PMC7293987 32606754

[63] Li C Wu H Yang Y Liu J Chen Z. Sesquiterpene lactone 6-o-angeloylplenolin reverses vincristine resistance by inhibiting YB-1 nuclear translocation in colon carcinoma cells. Oncol Lett (2018) 15:9673–80. doi: 10.3892/ol.2018.8592 PMC6004700 29928343

[64] Liu Y Chen X Liang H Zhang F Zhang B Jin J. Small compound 6-o-angeloylplenolin induces mitotic arrest and exhibits therapeutic potentials in multiple myeloma. PloS One (2011) 6:e21930. doi: 10.1371/journal.pone.0021930 21755010 PMC3130785

[65] Wang J Li M Cui X Lv D Jin L Khan M. Brevilin a promotes oxidative stress and induces mitochondrial apoptosis in U87 glioblastoma cells. OncoTargets Ther (2018) 11:7031–40. doi: 10.2147/OTT.S179730 PMC6198872 30410360

[66] You P Wu H Deng M Peng J Li F Yang Y. Brevilin a induces apoptosis and autophagy of colon adenocarcinoma cell CT26 *via* mitochondrial pathway and PI3K/AKT/mTOR inactivation. Biomed Pharmacother (2018) 98:619–25. doi: 10.1016/j.biopha.2017.12.057 29289836

[67] Wang Y Yu R Zhang J Zhang W Huang Z Hu H. Inhibition of Nrf2 enhances the anticancer effect of 6-o-angeloylenolin in lung adenocarcinoma. Biochem Pharmacol (2017) 129:43–53. doi: 10.1016/j.bcp.2017.01.006 28104435

[68] Chen X Du Y Nan J Zhang X Qin X Wang Y. Brevilin a, a novel natural product, inhibits janus kinase activity and blocks STAT3 signaling in cancer cells. PloS One (2013) 8:e63697. doi: 10.1371/journal.pone.0063697 23704931 PMC3660600

[69] Su M Chung H Li Y. 6-O-Angeloylenolin induced cell-cycle arrest and apoptosis in human nasopharyngeal cancer cells. Chemico Biological Interact (2011) 189:167–76. doi: 10.1016/j.cbi.2010.12.022 21192922

[70] Liu R Qu Z Lin Y Lee C Tai W Chen S. Brevilin a induces cell cycle arrest and apoptosis in nasopharyngeal carcinoma. Front Pharmacol (2019) 10:594. doi: 10.3389/fphar.2019.00594 31178739 PMC6544084

[71] Perez J Jayaprakasha G Valdivia V Munoz D Dandekar D Ahmad H. Limonin methoxylation influences the induction of glutathione s-transferase and quinone reductase. J Agric Food Chem (2009) 57:5279–86. doi: 10.1021/jf803712a PMC2730739 19480426

[72] Yang R Song C Chen J Zhou L Jiang X Cao X. Limonin ameliorates acetaminophen-induced hepatotoxicity by activating Nrf2 antioxidative pathway and inhibiting NF-κB inflammatory response *via* upregulating Sirt1. Phytomed: Int J Phytother Phytopharmacol (2020) 69:153211. doi: 10.1016/j.phymed.2020.153211 32259676

[73] Wang S Han X Yang Y Zhou C Luo D He W. Discovery of deoxylimonin δ-lactam derivative with favorable anti-inflammation and antinociception efficacy from chemical modified limonin/deoxylimonin analogs. Bioorganic Chem (2020) 100:103886. doi: 10.1016/j.bioorg.2020.103886 32371249

[74] Gao L Sang J Cao HJB. Limonin enhances the radiosensitivity of nasopharyngeal carcinoma cells via attenuating Stat3-induced cell stemness. Biomed Pharmacother (2019) 118:109366. doi: 10.1016/j.biopha.2019.109366 31545261

[75] Le Q Colevas A O’Sullivan B Lee A Lee N Ma B. Current treatment landscape of nasopharyngeal carcinoma and potential trials evaluating the value of immunotherapy. J Natl Cancer Institute (2019) 111:655–63. doi: 10.1093/jnci/djz044 PMC7962891 30912808

[76] Ke L Xia W Qiu W Huang X Yang J Yu Y. Safety and efficacy of lobaplatin combined with 5-fluorouracil as first-line induction chemotherapy followed by lobaplatin-radiotherapy in locally advanced nasopharyngeal carcinoma: preliminary results of a prospective phase II trial. BMC Cancer (2017) 17:134. doi: 10.1186/s12885-017-3080-4 28202000 PMC5311839

[77] Weng W Goel A. Curcumin and colorectal cancer: An update and current perspective on this natural medicine. Semin Cancer Biol (2020) 80:73–86. doi: 10.1016/j.semcancer.2020.02.011 32088363 PMC7438305

[78] Luo H Lu L Liu N Li Q Yang X Zhang Z. Curcumin loaded sub-30 nm targeting therapeutic lipid nanoparticles for synergistically blocking nasopharyngeal cancer growth and metastasis. J Nanobiotechnol (2021) 19:224. doi: 10.1186/s12951-021-00966-6 PMC8317404 34320999

[79] Momtazi-Borojeni AA Ghasemi F Hesari A Majeed M Caraglia M Sahebkar A. Anti-cancer and radio-sensitizing effects of curcumin in nasopharyngeal carcinoma. Curr Pharm Des (2018) 24:2121–8. doi: 10.2174/1381612824666180522105202 29788875

[80] Shao M Lou D Yang J Lin M Deng X Fan Q. Curcumin and wikstroflavone b, a new biflavonoid isolated from wikstroemia indica, synergistically suppress the proliferation and metastasis of nasopharyngeal carcinoma cells via blocking FAK/STAT3 signaling pathway. Phytomed: Int J Phytother Phytopharmacol (2020) 79:153341. doi: 10.1016/j.phymed.2020.153341 32992086

[81] Yao J Shen Q Huang M Ding M Guo Y Chen W. Screening tumor specificity targeted by arnicolide d, the active compound of centipeda minima and molecular mechanism underlying by integrative pharmacology. J Ethnopharmacol (2022) 282:114583. doi: 10.1016/j.jep.2021.114583 34487850

[82] Liu R Dow Chan B Mok DK Lee CS Tai WC Chen S. Arnicolide d, from the herb centipeda minima, is a therapeutic candidate against nasopharyngeal carcinoma. Molecules (2019) 24:1908. doi: 10.3390/molecules24101908 PMC6571971 31108969

